# The Mechanisms of Maize Resistance to *Fusarium verticillioides* by Comprehensive Analysis of RNA-seq Data

**DOI:** 10.3389/fpls.2016.01654

**Published:** 2016-11-04

**Authors:** Yanping Wang, Zijian Zhou, Jingyang Gao, Yabin Wu, Zongliang Xia, Huiyong Zhang, Jianyu Wu

**Affiliations:** College of Agronomy, Synergetic Innovation Center of Henan Grain Crops and National Key Laboratory of Wheat and Maize Crop Science, Henan Agricultural UniversityZhengzhou, China

**Keywords:** RNA-seq, maize, *Fusarium verticillioides*, ear rot, sHSPs, secondary metabolites, hormone signaling, PTI

## Abstract

*Fusarium verticillioides* is the most commonly reported fungal species responsible for ear rot of maize which substantially reduces grain yield. It also results in a substantial accumulation of mycotoxins that give rise to toxic response when ingested by animals and humans. For inefficient control by chemical and agronomic measures, it thus becomes more desirable to select more resistant varieties. However, the molecular mechanisms underlying the infection process remain poorly understood, which hampers the application of quantitative resistance in breeding programs. Here, we reveal the disease-resistance mechanism of the maize inbred line of BT-1 which displays high resistance to ear rot using RNA high throughput sequencing. By analyzing RNA-seq data from the BT-1 kernels before and after *F. verticillioides* inoculation, we found that transcript levels of genes associated with key pathways are dramatically changed compared with the control treatment. Differential gene expression in ear rot resistant and susceptible maize was confirmed by RNA microarray and qRT-PCR analyses. Further investigation suggests that the small heat shock protein family, some secondary metabolites, and the signaling pathways of abscisic acid, jasmonic acid, or salicylic acids (SA) may be involved in the pathogen-associated molecular pattern-triggered immunity against *F. verticillioides*. These data will not only provide new insights into the molecular resistant mechanisms against fungi invading, but may also result in the identification of key molecular factors associated with ear rot resistance in maize.

## Introduction

*Fusarium verticillioides* is an important pathogenic fungus, which causes stalk rot and ear rot in maize (White, 1999). *F. verticillioides* is the main maize pathogen and widespread in most of China. Symptoms of ear rot are related to genotype, environment and the degree of infection (Bacon et al., 2008). Another by-product of *F. verticillioides* infection is the production of deoxynivalenol and fumonisin, which causes food poisoning and cancer. Chemical and agronomic methods preventing ear rot are not very efficient because *F. verticillioides* can systemically infect maize without producing symptoms and can also spread from seeds to kernels (Munkvold and Desjardins, 1997; Wilke et al., 2007). For these reasons, host resistance is the most reliable and economical way to reduce the damage caused by ear rot.

Progress in resistance breeding will be hastened by the analysis of new and consistent quantitative trait loci (QTLs) for Fusarium ear rot resistance and fumonisin accumulation, as well as by a deeper knowledge of the genetic mechanisms underlying maize–*F. verticillioides* interactions. The genetic mechanisms for *F. verticillioides* resistance had some reported (Yuan et al., 2013; Ridenour et al., 2016), however, the QTLs associated with *F. verticillioides* resistance usually have small effects and are not stable in different populations (Pérez-Brito et al., 2001; Robertson-Hoyt et al., 2006; Ding et al., 2008; Xiang et al., 2010; Li et al., 2011; Mesterházy et al., 2012). In our previous research, a stable QTL on chromosome 4 was detected by two populations in different environments and verified by near-isogenic lines (Chen et al., 2012). The common resistant parent (BT-1) of two populations showed strengthened resistance, close to immunity, to the ear rot and stalk rot caused by *F. verticillioides*, and thus is an ideal material for study of maize resistance against *F. verticillioides*.

Extensive studies on model species have clarified some crucial events in plant resistance. Plants have evolved two defense mechanisms against pathogen invasion that involve different strategies for detecting pathogens. On the extracellular face of the host cell, pathogen-associated molecular patterns are recognized by pattern recognition receptors, and their subsequent stimulation leads to pathogen-associated molecular pattern-triggered immunity (PTI) (Dodds and Rathjen, 2010). PTI induces mitogen-activated protein kinases and calcium signaling, the transcription of pathogen-responsive genes, the production of reactive oxygen species and the deposition of callose to reinforce the cell wall at sites of infection (Nürnberger et al., 2004). In addition, plants have evolved a more specialized defense mechanism toward successful pathogens, effector-triggered immunity (ETI), which acts largely inside the cell and involves the recognition of pathogen-delivered effectors, which contribute to pathogen virulence by interacting with plant resistance proteins. ETI is an accelerated and amplified PTI response, resulting in disease resistance and, usually, a hypersensitive cell death response at the infection site. Following the early signaling events activated by pathogen attack, elicitor signals are often amplified through the generation of secondary signal molecules, such as salicylic acid (SA), ethylene (ET) and jasmonic acid (JA). In addition, the defense response in plant–fungal interactions is also closely related to the accumulation of many secondary metabolites (Sekhon et al., 2006; Lanubile et al., 2014).

Small heat shock proteins (sHSPs), or HSP20s, ranging in size from 12 to 43 kDa, have variable sequences and are characterized by a conserved region of approximately 90 residues that form an α-crystallin domain (Caspers et al., 1995). These proteins form large oligomers and perform their ATP-independent chaperone function *in vitro* by binding to (partially) denatured proteins (Lee et al., 1995; Helm et al., 1997; Kirschner et al., 2000). *In vivo*, sHSP20s confer a protective function by preventing the unfolding or disassembly of other proteins (van Montfort et al., 2001). sHSP20s probably maintain denatured proteins in a folding-competent state to allow for the subsequent ATP-dependent disaggregation by the HSP70/90 chaperone system (Kotak et al., 2007; Liberek et al., 2008). Furthermore, the sHSPs are related to biotic or abiotic stress responses in different plant species (Van Ooijen et al., 2010; Lopes-Caitar et al., 2013).

In this research, we studied the transcriptional alteration of the resistant maize inbred line BT-1 in early and later infection stages in response to *Fusarium verticillioides*, and evaluated the transcriptional differences in some key genes between BT-1 and the susceptible inbred line ‘N6’ by RNA microarray and qRT-PCR. The results showed that the PTI process and signal pathways of abscisic acid (ABA), JA, and SA were respectively activated in the later stage after inoculation with BT-1, and that sHSP families as well as some secondary metabolites may also be involved in this process, and thus leading to variable resistance levels in different maize materials. Taken together, these data provides novel and valuable information that will help understand the distinctive resistance mechanism in plants against fungal invasions and locate candidate genes related to maize resistance against *F. verticillioides*.

## Materials and Methods

### Plant Materials and Growth Conditions

The resistant inbred line, BT-1, and susceptible inbred line, N6, were grown at the Zhengzhou Experiment Station (34°510′N 113°350′E) in June 2013 and June 2014. The resistant line BT-1 which was one of the parental lines for the elite hybrid Guoshenyu2005026 was originated from a cross between Thailand Suwan and inbred line 8085. The susceptible parental line N6 was an improved inbred line from huangzaosi which has high combining ability, good plant type, short growth period and high stress resistance with weak disease resistance. BT-1 and N6 have been identified as one of the best materials which showed the highest resistant and susceptible to Furiasum ear rot *F. verticillioides*, respectively (Chen et al., 2012; Yuan et al., 2013). The maize kernels were inoculated with *F. verticillioides*, and with water as the control, on day 15 after pollination. The kernels around the inoculation spots were collected at different times and placed in liquid nitrogen. The kernel samples of BT-1 collected at 0, 24, and 240 h after inoculation were used for RNA-seq and RNA microarray analyses in 2013. The kernel samples of BT-1 and N6 at 0, 24, and 240 h after inoculation were used for the qRT-PCR analysis in 2014. The RNAs were extracted from three mixed independent samples and each one comprised 10 kernels around the inoculation spot.

### Inoculation Method

The pathogen was isolated from naturally infected kernels using the single-spore isolation method described by Ho and Ko (1997). To prepare the field inoculums, sterilized maize grains were used as the substrate to produce large quantities of spores. The washed maize grains were boiled for 10 min, air-dried for 30 min, and placed in 1 L jars (3/4-filled). The jars were then sterilized in the autoclave. After naturally cooling to room temperature, the grains were inoculated in a biosafety cabinet with 5 mL of *F. verticillioides* spore suspension, which had been washed from a Petri dish containing a pure culture of the fungus. The jars were incubated at 26–30°C for 2–3 weeks. Before the field was inoculated with ear rot, the maize grains were removed from the jars and placed in a clean container, 1 L of sterile water was added at 4°C and mixed well, the mixture was filtered using two layers of gauze placed in a funnel to collect the concentrated spore solution, and the spore solution was diluted to 1 × 10^6^ spores/mL for field inoculations. The inoculation was performed using the sponge and nail punch method (Drepper and Renfro, 1990) 15 days after self-pollination. A clear plastic bag was used to cover the ear to maintain humidity and avoid further allo-infection after inoculation.

### cDNA Library Preparation and Transcriptome Sequencing

Total RNA was extracted from the indicated samples using an RNAisomate RNA Easyspin Isolation System (Aidlab Biotech, Beijing, China) according to the manufacturer’s instructions. The quality of RNA was verified using a 2100 Bioanalyzer (Agilent Technologies, Santa Clara, CA, USA). To prepare cDNA, we used a pooled RNA mixture containing 60 μg RNA from each sample. Illumina sequencing was conducted using the Solexa mRNA-seq platform using the manufacturer’s instructions (Illumina, San Diego, CA, USA). Briefly, we used magnetic beads to isolate total RNA from *Zea mays* kernels. Second-strand cDNA was synthesized using appropriate buffers, dNTPs, RNase H, and DNA polymerase I. ShoBiotechnology Corporation (Shanghai, China^1^) prepared the rt fragments, which were depurated with a QiaQuick PCR extraction kit (Qiagen, Hilden, Germany), and resolved with an elution buffer for end repair and by the addition of poly(A). For PCR amplification, we selected suitable fragments as templates based on the results of agarose gel electrophoresis. The library was sequenced using an Illumina HiSeq^TM^ 2500 (Paired, 100 nt). Because raw reads produced from sequencing machines contain low-quality reads that negatively affect subsequent bioinformatics analyses, we discarded reads with adapters, those with more than 5% unknown nucleotides, and those of low quality (≤20% of the bases with a quality score ≤10) using an in-house Perl script. The average proportion of clean reads in each sample was 91.88–93.48%. The clean reads were used for further analyses.

### Bioinformatics Analysis

The clean reads were aligned to the maize B73 reference genome (ZmB73_RefGen_v3^2^) using TopHat v2.0.6 (Trapnell et al., 2009), given the parameters derived mean read spacer size (110 bp) and its standard deviation (100 bp). Alignments were processed with Cufflinks 2.0.2 (Trapnell et al., 2010) to assemble transcript isoforms and quantify expression values, such as fragments per kilobase of exon model per million mapped reads (FPKM) of known and novel genes using the maize working gene set as the reference annotation (AGPv3^2^) and to guide the RABT assembly using default parameters. A differential expression analysis was performed with the DESeq package (Anders and Huber, 2010), with a False Discovery Rate (FDR) threshold of 0.05 and a Log2 fold change. Cang Sequences of differentially expressed genes (DEGs) were compared with the NCBI non-redundant (NR) database using the BLAST algorithm with an *E*-value of 10^-3^ and were functionally annotated using Blast2GO, which assigned GO terms and the metabolic pathway in the Kyoto Encyclopedia of Genes and Genomes (KEGG) to the query sequences. The clustering of FPKM expression values of DEGs was performed using a Euclidean distance measure with complete linkage.

### Data Deposition

Transcriptome sequence data from this article can be found in the National Center for Biotechnology Information (NCBI) Sequence Read Archive under accession numbers SRP077851.

### Microarray Hybridization and Data Analysis

A 2-mg aliquot of total RNA was used to synthesize fluorophore-labeled cRNA using cyanine 3-CTP (One-color microarray-based gene expression analysis protocol, Version 5.5; Agilent Technologies). Samples were purified using a Qiagen RNeasy Kit (Qiagen, Hilden, Germany). The quality of cyanine-labeled cRNA samples, including yield, concentration, amplification efficiency and abundance of the cyanine fluorophore, was determined using an ND-1000 spectrophotometer at OD_260_ and OD_550_. Once the concentration had been determined, cyanine-labeled cRNA fragmentation and microarray slide hybridization followed (Agilent Technologies). After hybridization, the microarray slides were washed according to the standard Agilent protocol and scanned on a microarray at 550 nm using an Agilent microarray scanner (G2505B). The information procured by the scanner was loaded into the image analysis program ‘Feature Extraction version 9.5’ to establish standard data for the statistical analysis, and all of the microarray slides were checked for background evenness. Datasets were further analyzed according to published procedures, which consisted of one-color experimental methods and utilized gProcessedSignal values determined by Agilent Feature Extraction software, including aspects of the signal-to-noise ratio, spot morphology and homogeneity (Patterson et al., 2006; Gobert et al., 2009). If the processed signal intensity was less than twofold the value of the processed signal error, then the features were deemed ‘Absent.’ If the measured intensity was at a saturated value, or if there was a substantial amount of variation in the signal intensity within pixels of a particular feature, then the features were deemed ‘Marginal.’ Otherwise, the features were deemed ‘Present.’ Data points were included only if ‘Present’ or ‘Marginal,’ and probes were retained if all of the data points were ‘Present.’

## Results

### Transcriptome in BT-1 Kernels Responding to the Pathogen of *F. verticillioides*

To understand the molecular basis of resistance to *F. verticillioides* in maize, we analyzed the transcriptomic alterations of BT-1, one of resistance inbred parental lines, in response to *F. verticillioides* infection. We chose five seed samples, including pretreatments, treatment 1 day post-inoculation (dpi) or 10 dpi with *F. verticillioides* or water, and extracted the total RNA for deep sequencing. The raw reads from sequencing were filtered and mapped to the maize genome. The clean reads filtered from the raw reads of five samples were all greater than 90%. On average, 79.7% of the total reads mapped to the maize inbred B73 reference genome sequence (**Table 1**), with most of the reads mapping to exons and only a small portion mapping to introns or intergenic regions.

**Table 1:** Clean reads mapped to the maize B73 genome.

Samples	All reads	Mapped reads	Mapped pair reads	Mapped broken-pair reads	Mapped unique reads	Mapped multi reads	Mapping ratio
0d	37,376,272	29,534,452	26,620,752	2,913,700	24,542,759	4,991,693	79.01%
T_1d	35,381,110	29,021,440	26,399,806	2,621,634	24,508,069	4,513,371	82.03%
T_10d	40,591,734	32,721,193	29,398,812	3,322,381	27,727,168	4,994,025	80.61%
M_1d	40,089,944	32,437,937	29,239,360	3,198,577	27,004,643	5,433,294	80.91%
M_10d	38,083,336	28,961,951	26,040,674	2,921,277	24,090,988	4,870,963	76.05%


*0d, the untreated sample. T_1d, the sample 1 day post inoculation by *F. verticillioides*. T_10d, the sample 10 day post inoculation by *F. verticillioides*. M_1d, the sample 1 day post treatment by water. M_10d, the sample 10 day post treatment by water.*

To further solid the RNA-seq data, we performed a parallel test of microarray hybridizations using the same batch of samples. We chose the normalized expression of single-probe genes, which had ‘Present’ quality characteristics in five samples, to compare with the expression levels of same genes in RNA-seq dataset. As shown in **Supplementary Figure S1**, the results generated by the two methods agreed well.

### Analysis of Genes Expressed in Maize Kernels Specifically Responsive to *F. verticillioides*

**FIGURE 1:**
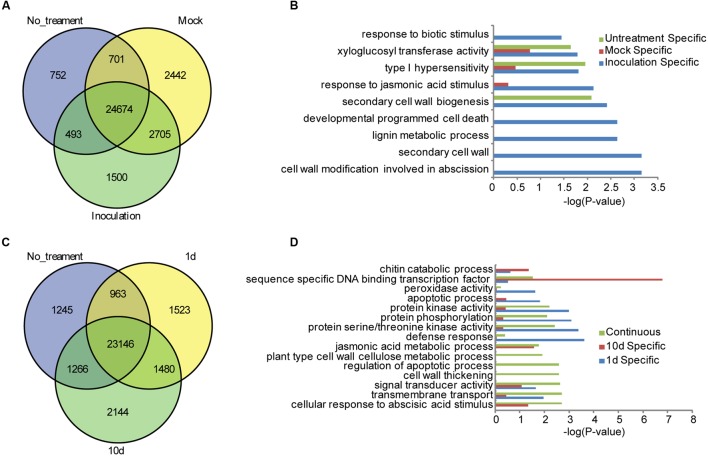
**Specifically expressed genes in the different tested samples of BT-1.** **(A)** Venn diagrams of expressed genes in no-treatment (No_treatment), water (Mock) and *F. verticillioides*-inoculated (Inoculation) test samples. **(B)** Enriched GO terms in the Biological Process category that are associated with the specifically expressed genes in no-treatment (Untreatment Specific), water (Mock Specific) and *F. verticillioides*-inoculated (Inoculation Specific) test samples. Abscissae represent GO enrichment -LOG_10_ FDR values. **(C)** Venn diagrams of expressed genes in no-treatment (No_treatment), 1 day post inoculation (1d) and 10 days post inoculation (10d) test samples. **(D)** Enriched GO terms in the Biological Process category that are associated with the specifically expressed genes in *F. verticillioides*-inoculated (Inoculation Specific) test samples. Genes specifically expressed in 1 day post inoculation (1d), 10 days post inoculation (10d) test samples and genes expressed in the two samples (Continuous) were enriched respectively. Abscissae represent GO enrichment -LOG_10_ FDR values.

To facilitate the global identification of responsive genes in the resistant maize inbred line of BT-1 treated with *F. verticillioides*, gene expression levels were quantified using maize B73 as the reference genome, and the abundance of each transcript was expressed as FPKM as implemented by Cufflinks (Trapnell et al., 2010). Genes were defined as DEGs with an FDR threshold of 0.05 and a Log2 fold change ≥ 1(Anders and Huber, 2010). After data processing, a total of 26,621 genes were identified to be expressed in the non-treated sample and 29,372 genes in the water-treated sample expressed at a FPKM > 1. By contrast, 30,522 genes were identified to be expressed in the sample inoculated with *F. verticillioides*. Comparative analysis further revealed 24,674 common genes in all three cases, of which 752 genes were specifically expressed in non-treated samples, 2,442 genes in the water-treated sample, and 1,500 genes in the sample inoculated with *F. verticillioides*, respectively (**Figure 1A**; **Supplementary Table S1**). Gene Ontology (GO) analysis of specifically expressed genes after inoculation with *F. verticillioides* revealed these genes preferentially associated with GO terms such as cell wall modification involved in abscission (GO:0009830), secondary cell wall (GO:0009531), lignin metabolic process (GO:0009808), developmental programmed cell death (GO:0010623), secondary cell wall biogenesis (GO:0009834), response to JA stimulus (GO:0009753), type I hypersensitivity (GO:0016068), xyloglucan:xyloglucosyl transferase activity (GO:0016762), response to biotic stimulus (GO:0009607) (**Figure 1B**). Together, these results indicate that distinctive resistance pathways will be activated in the BT-1 kernel after inoculation with *F. verticillioides*, such as hypersensitivity, JA signal pathway, and cell wall thickening against fungal invasions.

A total of 27,112 genes were identified to be expressed at 1 dpi while 28,036 genes at 10 dpi. Among these genes, 1,523 and 2,144 genes were specifically expressed at 1 dpi and 1 10 dpi, respectively. Interestingly, 1,480 genes were expressed in both 1 and 10 dpi samples but no in the non-treatment sample, suggesting that these genes are specifically responsive to *F. verticillioides* (**Figure 1C**; **Supplementary Table S1**). Global analysis showed that these 1,480 genes are preferentially associated with different GO terms such as cellular response to abscisic acid stimulus (GO:0071215), transmembrane transport (GO:0055085), signal transducer activity (GO:0004871), cell wall thickening (GO:0052386), regulation of apoptotic process (GO:0042981), plant-type cell wall cellulose metabolic process (GO:0052541), JA metabolic process (GO:0009694). As to the 1,523 of specifically expressed genes in the 1 dpi sample, they were significantly enriched in terms of defense response (GO:0006952), protein serine/threonine kinase activity (GO:0004674), protein phosphorylation (GO:0006468), protein kinase activity (GO:0004672), apoptotic process (GO:0006915), signal transducer activity (GO:0004871), peroxidase activity (GO:0004601), indicating that the PTI pathway had been activated and multiple members of protein kinases were induced at 1 dpi. GO analysis were further performed for the 2,144 of specifically expressed genes in the 10 dpi sample, and found that they were preferentially associated with sequence-specific DNA binding transcription factor activity (GO:0003700), chitin catabolic process (GO:0006032) (**Figure 1D**), indicating that a large number of TFs may associated with activation of downstream disease-resistant gene at 10 dpi. These results indicated that protein kinases were quickly induced to activate the PTI defense responses after *F. verticillioides* invasion while the TFs associated with disease resistance were robustly expressed and activated downstream genes in the late stage of invasion. However, cross-talk of various hormone signaling pathways and the reactions of cell wall thickening and apoptosis induced by hypersensitivity, remained to be activated through the whole process of *F. verticillioides* invasion.

### Temporal-Specific Genes during Seed Development in Maize

**FIGURE 2:**
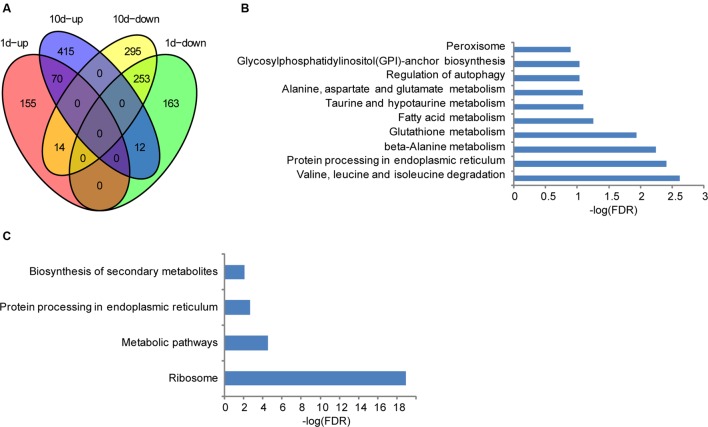
**Differentially expressed genes in different seed development stages of BT-1.** **(A)** Venn diagrams of differentially expressed genes in water treatment samples (vs. no treatment samples). 1d-up: the genes up-regulated in 1d post treat. 1d-down: the genes down-regulated in 1d post treat. 10d-up: the genes up-regulated in 10d post treat. 10d-down: the genes down-regulated in 10d post treat. **(B)** Enriched pathways that are associated with the differentially expressed genes in 1 day post water treatment samples. Abscissae represent KEGG Pathway enrichment -LOG_10_ FDR values. **(C)** Enriched pathways that are associated with the differentially expressed genes in 10 days post water treatment samples. Abscissae represent KEGG Pathway enrichment -LOG_10_ FDR values.

Although the seeds showed no visible morphological changes after treated with water for 1 or 10 days compared with the pre-treatment ones, distinctive levels of gene expression were observed as mentioned above, suggesting that altered genes may be related to seed development in maize. To further clarify their biological functions, KEGG analyses were performed. Compared to the water-treated 1 dpi sample, 239 and 428 genes were significantly up- and down- regulated in the treatment sample, respectively (**Figure 2A**). Regarding annotated pathways, those associated with the nutritional materials metabolism are among the most significantly enriched, such as valine, leucine, and isoleucine degradation, protein processing in endoplasmic reticulum, β-Alanine metabolism, glutathione metabolism, fatty acid metabolism, taurine and hypotaurine metabolism, Alanine, aspartate and glutamate metabolism, regulation of autophagy, glycosylphosphatidylinositol (GPI)-anchor biosynthesis, and peroxisome (**Figure 2B**). In addition, 497 and 562 genes were significantly up- and down- regulated in the treated samples compared with the water-treatment sample at 10 dpi, respectively. They are preferentially associated with the pathways of nutritional materials and energy metabolism, such as ribosome, metabolic pathways, protein processing in endoplasmic reticulum, biosynthesis of secondary metabolites, and valine, leucine, and isoleucine degradation (**Figure 2C**). These data indicate that the specifically expressed genes in different development stage are only relevant to metabolites storage during seed development, and thus facilitate uncovering the molecular mechanisms underlying the resistance of *F. verticillioides* in BT-1 maize.

### Characteristics of Genes Responsive to *F. verticillioides* in Corn Kernels

**FIGURE 3:**
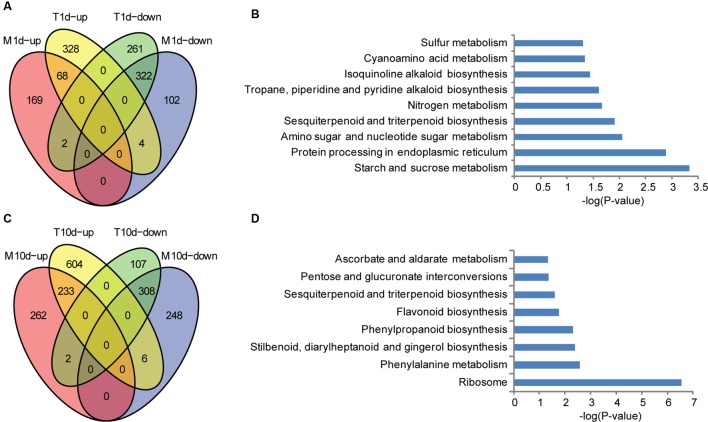
**Specific up- and down-regulated genes in BT-1 maize at different times after water (M) or *F. verticillioides* (T) inoculation.** **(A)** Venn diagrams of differentially expressed genes in water treatment samples (vs. no treatment samples). T1d-up: the genes up-regulated in 1dpi. T1d-down: the genes down-regulated in 1 dpi. M1d-up: the genes up-regulated in 1d post water treat. M1d-down: the genes down-regulated in 1d post water treat. **(B)** Enriched pathways that are associated with the specific regulated genes in 1 dpi samples. Abscissae represent KEGG Pathway enrichment -LOG_10_ *P*-values. **(C)** Venn diagrams of differentially expressed genes in water treatment samples (vs. no treatment samples). T10d-up: the genes up-regulated in 10 dpi. T10d-down: the genes down-regulated in 10 dpi. M10d-up: the genes up-regulated in 10 days post water treat. M10d-down: the genes down-regulated in 10 days post water treat. **(D)** Enriched pathways that are associated with the specific regulated genes in 10 dpi samples. Abscissae represent KEGG Pathway enrichment -LOG_10_ *P*-values.

To address the resistance mechanisms of BT-1 in more details, we focused on the genes specifically influenced by *F. verticillioides*. First, expressed genes from water-treated samples were compared with those from *F. verticillioides*-inoculated at 1 dpi. 400 and 585 genes were found to be significantly up- and down- regulated in the inoculated samples, respectively. As shown in **Figure 3A** (**Supplementary Table S2**), 332 of 400 up-regulated genes and 263 of the 585 down-regulated genes were specific for *F. verticillioides* inoculation, respectively. These genes were then employed for the KEGG enrichment analysis. Several representative pathways are among the most significantly enriched, such as starch and sucrose metabolism (KO:zma00500), protein processing in endoplasmic reticulum (KO:zma04141), amino sugar and nucleotide sugar metabolism (KO:zma00520), sesquiterpenoid and triterpenoid biosynthesis (KO:zma00909), isoquinoline alkaloid biosynthesis (KO:zma00950), cyanoamino acid metabolism (KO:zma00460), and Sulfur metabolism (KO:zma00920) (**Figure 3B**). Interestingly, terpenes and endoplasmic reticulum protein processing was identified to be preferentially associated with ectopically expressed genes, suggesting that it may be involved in early defense responses against fungal infections.

Second, the expressed genes were compared between water-treated samples and *F. verticillioides*-inoculated at 10 dpi. The treated samples had 843 and 417 genes that were significantly up- and down- regulated, respectively. 610 of the up-regulated genes and 109 of the down-regulated genes were specifically responsive to *F. verticillioides* inoculation, respectively (**Figure 3C**; **Supplementary Table S2**). Regarding to annotated pathways, the specific genes were significantly associated with the following processes, especially antibiotic and terpenoid biosynthetic processes, such as ribosome (KO:zma03010), phenylalanine metabolism (KO:zma00360), stilbenoid, diarylheptanoid and gingerol biosynthesis (KO:zma00945), phenylpropanoid biosynthesis (KO:zma00940); flavonoid biosynthesis (KO:zma00941), sesquiterpenoid and triterpenoid biosynthesis (KO:zma00909), pentose and glucuronate interconversions (KO:zma00040), and ascorbate and aldarate metabolism (KO:zma00053) (**Figure 3D**). These data indicates that genes involved in biosynthesis of secondary metabolites that are further transformed to antibacterial compounds are preferentially induced at the late stage of *F. verticillioides* infection and thus likely leading to resistant characteristics in BT-1 maize.

**FIGURE 4:**
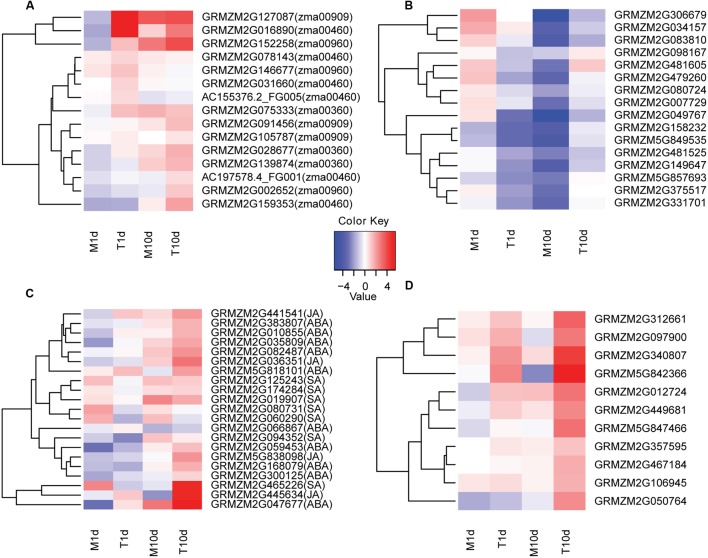
**Clustering and heat maps of representative genes in indicated samples of BT-1.** **(A)** The expression levels of several secondary metabolite-related genes in different samples. The corresponding KEGG pathways are in parentheses. **(B)** The expression levels of sHSPs family genes in different samples. **(C)** The expression levels of genes related to hormone signaling pathways in different samples. The corresponding hormone pathways are in parentheses. **(D)** The expression levels of genes related to pathogen identification signaling pathways in different samples. The color scale indicates the fold changes of gene expression. Sample names are displayed below the heat maps. M1d: 1 day post water treatment samples. M10d: 10 day post water treatment samples. T1d: 1 day post inoculation samples. T10d: 10 day post inoculation samples. A fold change of ≥ 1 is shown in red (increased transcript abundance), a fold change of ≤-1 is shown in blue (decreased transcript abundance), and no change is indicated in white.

Furthermore, a detailed investigation of the DEGss in representative pathways revealed that genes involved in biosynthesis of secondary metabolites may play crucial role in the disease resistance of the BT-1 maize. For instance, expression levels of the key gene of *GRMZM2G146677* encoding aspartate aminotransferase as well as another two genes of *GRMZM2G002652* and *GRMZM2G152258* involved in the tropine biosynthesis pathway were significantly increased in the samples inoculated with *F. verticillioides* either at 1 or 10 dpi (**Figure 4A**; **Supplementary Figure S2**). Analyzing the up-regulated genes involved in cyanoamino acid metabolism, transcripts levels of six genes that directly contribute to hydrogen cyanide biosynthesis were dramatically increased in *F. verticillioides*-inoculated samples (**Figure 4A**; **Supplementary Figure S3**). However, they remained unchanged in the water-treated samples, indicating that enhanced biosynthesis of hydrogen cyanide occurs in the grains after *F. verticillioides* infection.

In terms of phenylalanine metabolism (KO:zma00360), three genes of GRMZM2G139874, GRMZM2G028677 and GRMZM2G075333 that fulfill trans-cinnamate 4-monooxygenase and 4-coumaric acid coenzyme A ligase functions were significantly increased in *F. verticillioides*-inoculated samples compared with the water-treated samples over the same time period (**Figure 4A**; **Supplementary Figure S4**). Therefore, after *F. verticillioides* inoculation, enhanced phenylpropanoic acid metabolism will promote the formation of some important disease-resistant secondary metabolites, such as lignin, coumaric acid and flavonoids. Similarly, genes involved in phenylpropanoid and flavonoid biosynthetic processes were also dramatically induced in the *F. verticillioides*-inoculated samples, coinciding well with the observations mentioned above.

It is worth noting that specifically up- and down- regulated genes at both 1 and 10 dpi were significantly enriched in the sesquiterpenoid and triterpenoid biosynthesis processes. Further analysis revealed that the terpene synthase 6 gene of GRMZM2G127087 at 1 dpi as well as two genes of GRMZM2G091456 and GRMZM2G105787 at 10 dpi both encoding squalene epoxidases were specifically up-regulated in the *F. verticillioides*-inoculated samples (**Figure 4A**; **Supplementary Figure S5**). Thus, it is likely that increased accumulation of macrocarpa and triterpenoid confers BT-1 the capability against *F. verticillioides*.

It is intriguing that the protein processing in the endoplasmic reticulum is among the most significantly enriched pathways comprising the *F. verticillioides*-responsive genes at 1 dpi. Eight genes involved in this process were dramatically increased in the *F. verticillioides*-treated samples compared with the water-treated ones, of which six genes encode sHSP proteins besides *GRMZM2G078526* and *GRMZM2G019236* for zinc finger proteins. It has been reported that sHSP proteins are particularly associated with biotic or abiotic responses in plants (Van Ooijen et al., 2010; Lopes-Caitar et al., 2013). Thus, we specifically focused on the sHSP-related processes (**Supplementary Figure S6**). Of the 21 sHSP genes expressed in the *F. verticillioides-*treated samples, the expression levels of 16 genes displayed a fluctuation pattern that they were rapidly declined at 1 dpi compared with pre-treatment and then went up at 10 dpi. By contrast, the sHSP levels declined linearly either at 1 or 10 dpi in the water-treated samples compared with pre-treatment (**Figure 4B**). These data indicate that sHSP genes associated with protein processing may be specifically involved in resistance responsive regulation of BT-1 maize response to *F. verticillioides*. Collectively, analyses of DEGss reveal that regulation of multiple metabolisms is involved in the resistant trait of BT-1 maize against *F. verticillioides*, especially through manipulating the secondary metabolites and sHSP-associated protein processing.

### Regulation of Specific Signaling Pathways Responding to *F. verticillioides* in BT-1 Maize

Plants have evolved two defense mechanisms against pathogen invasion, either the PTI or ETI mechanism comprising extensive signal transductions (Dodds and Rathjen, 2010). Thus, we also assessed the signal transduction pathways associated with plant hormones and pathogen recognition in the current dataset. As to plant hormone signal transduction (KO:zma04075), a large number of genes involved in ABA, JA, or SA signaling pathways were significantly increased in the BT-1 kernels after *F. verticillioides* infection (**Figure 4C**; **Supplementary Figure S7**). In terms of plant-pathogen interaction, many genes involved in the pathogen recognition process of the PTI mechanism were dramatically induced after *F. verticillioides* treatment, and thus led to hypersensitivity, enhanced formation of secondary cell wall, and induction of resistance genes (**Figure 4D**; **Supplementary Figure S8**). This observation indicates that after *F. verticillioides* infection, BT-1 maize activates systemic acquired resistance immune reaction and specific hormone signaling pathways via the PTI mechanism and thus resistant capability.

### Expression of Representative Genes in Resistant (BT-1) and Susceptible (N6) Maize

**FIGURE 5:**
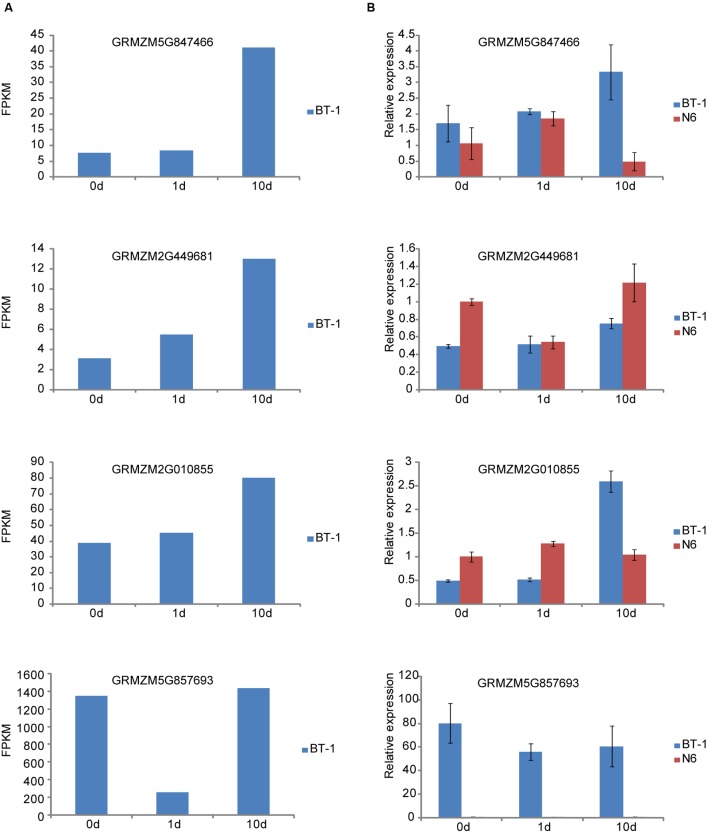
**Quantitive RT-PCR validation of representative genes in resistant (BT-1) and susceptible (N6) maize.** **(A)** The RNA-seq results of four genes associated with PTI process, ABA signal and Hsp20 in BT-1 (resistant line). **(B)** The qRT-PCR results of the four genes in BT-1 and N6 (susceptible lien). Data are means ± SD of three biological repeats.

To confirm the results presented above, we determined expression changes of several representative genes responsive to *F. verticillioides*. Four genes were selected for further analysis, in which GRMZM5G847466 (*CaM*) and GRMZM2G449681 (*FPKM53*) are associated with the PTI mechanism, and another two genes of GRMZM2G010855 for protein phosphatase 2C and GRMZM5G857693 for HSP20 are involved in ABA and sHSP pathways, respectively. We first examined the expression of the four genes in the sequencing dataset (**Figure 5A**), which agreed well with the results from RT-qPCR analysis except for the HSP20 gene (**Figure 5B**). Next, we performed comparison analysis between the resistant (BT-1) and susceptible (N6) maize (**Figure 5B**). Expression levels of *CaM* and *FPKM53* were persistently induced in BT-1 after *F. verticillioides* inoculation. However, they displayed distinctive expression pattern in N6. As shown in **Figure 5B**, *CaM* was induced at 1 dpi and then reduced at 10 dpi compared with pre-treatment, and the expression pattern of *FPKM53* was completely contrary to the *CaM*. The transcripts level of *GRMZM2G010855* was moderately changed in N6 but greatly increased in BT-1 after *F. verticillioides* inoculation. Interestingly, HSP20 expression was hardly detected in all three tested N6 samples while showed significantly decrease compared with pre-treatment in BT-1. Taken together, these results suggest that the difference between BT-1 and N6 resistance to *F. verticillioides* is associated with the PTI process as well as ABA and sHSP signaling pathway, but this requires further research.

### DEGs Responsive to *F. verticillioides* Are Enriched in Known QTLs

**FIGURE 6:**
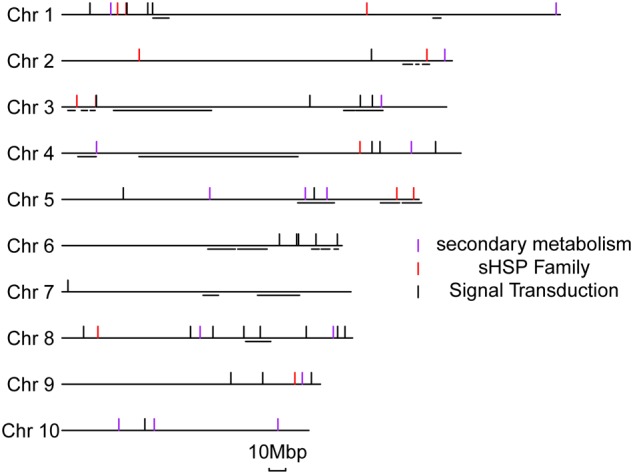
**Distribution of representative DEGs located in the metaQTL for *Fusarium verticillioide* ear rot.** On the left side, the number and relative lengths of the 10 maize chromosomes are indicated. The short horizontal lines represent the locations of QTLs of anti-ear rot from the metaQTL of Xiang. The short vertical lines represent the different types of specific disease-resistant response genes.

We were able to map the DEGs at 1 or 10 dpi to (592 and 716 genes, respectively) to known QTLs related to ear rot resistance in the maize genome (**Supplementary Figure S9**; Xiang et al., 2010). One important piece of evidence is the fact that those DEGs are not randomly distributed but preferentially clustered in specific chromosomal regions which are particularly associated with the known resistance QTLs. Among the DEG-related QTLs, many are well characterized, including an early expressed QTL on chromosome 3 and a late expressed QTL on chromosome 6. The DEGs of sHSP proteins, secondary metabolisms, and signal transductions were specifically aligned to previous QTLs locations in details (**Figure 6**). We found that 4 of 15 genes involved in secondary metabolisms, 5 of 16 sHSP genes, and 10 of 36 genes involved in signal transduction processes are respectively overlapped or very close to the known resistance QTLs in maize. The potential association between DEGs and QTLs further supports the resistant mechanisms of BT-1 maize against *F. verticillioides* we proposed in this work.

## Discussion

### Feasibility Study on Resistance Mechanism of Maize to *F. verticillioides* by High Throughput RNA Sequencing

Technological advances have made deep RNA sequencing feasible, expanding our view of the transcriptome (Wang et al., 2008) and promising to permit quantitative profiling with large dynamic range (Mortazavi et al., 2008). In current work, we attempted to explore the resistance mechanism to *F. verticillioides* in maize by analysis of differential genes via high throughput RNA sequencing. However, it has been shown that RNA-seq data have measurement noise which is a direct consequence of the random sampling process inherent to the assay. To assess the RNA-seq data presented here, we first performed microarray experiments using the same batch of samples as in the RNA-seq. Comparisons of the data from the two different platforms revealed a good correlation (**Supplementary Figure S1**). Next, expression levels of several representative genes by RT-qPCR are well consistent with the ones in RNA-seq data (**Figure 5**). Thus, RNA-seq data in this study are accurate and reproducible.

### sHSP Proteins As Well As Secondary Metabolites Play Crucial Roles in the Resistance to *F. verticillioides* in Maize

Genes associated with both seeds development and resistance regulation had been identified according to the RNA sequencing data in this study. Analysis of DEGss in the water-treated samples revealed that they were significantly enriched in nutrient storage-related processes in terms of KEGG, which is consistent with our knowledge about the grain filling process in maize as well as the results by Li et al. (2014). We also found that genes involved in signal transduction or secondary metabolism were particularly responsive to *F. verticillioides*, especially those encoding sHSP proteins, which are supported by a previous study of *F. verticillioides* caused ear rot in maize (Lanubile et al., 2014).

In the present study, 16 genes from the sHSP family were rapidly down-regulated in maize after being inoculated with *F. verticillioides*, suggesting their potential roles in the *F. verticillioides*-induced resistance. It was reported that the members of sHSP family were related to abiotic stress resistance (Sarkar et al., 2009; Guo et al., 2015), but few effort has been devoted to characterizing their biological functions in response to biotic stress in plants. A total of 51 genes encode sHSP proteins in soybean genome, six of which were responsive to the pathogen of *Meloidogyne javanica* (Lopes-Caitar et al., 2013). Regarding the disease-resistance function, Van Ooijen et al. (2010) found that the RSI2 gene belonging to Hsp20 family is required for the stability and disease-resistant function of I-2 protein in potato. Our data show that 16 of 21 genes of the sHSP family were significantly down-regulated in the resistant maize inbred BT-1 line after inoculation with *F. verticillioides*, which clearly suggests a primary role of the sHSP proteins in resistance to fungal infection in maize.

Interestingly, a number of genes involved in secondary metabolites biosynthesis are specifically responsive to *F. verticillioides* inoculation in BT-1 kernels. In support of this observation is the similar results from a previous study (Lanubile et al., 2014), in which the processes of Shikimate biosynthesis (KO:00400), phenylpropanoid biosynthesis (KO:00940), flavonoid biosynthesis (KO:00941), terpenoid biosynthesis (KO:00900), and diterpenoid biosynthesis (KO:00904) were particularly affected by *F. verticillioides* inoculation. In our study, phenylpropanoid and flavonoid biosynthesis were also significantly increased in response to *F. verticillioides*. The genes for *trans*-cinnamic acid 4 monooxygenase and 4-coumaric acid coenzyme A ligase that function upstream the phenylalanine metabolism (KO:zma00360) process were significantly induced. These results demonstrate that biosynthesis of benzenepropanoic acid is enhanced in BT-1 after *F. verticillioides* inoculation to produce more disease-related secondary metabolites, such as lignin, coumaric acid, and flavonoids. Additionally, the terpenoids process showed a similar trend. Compared to the Lanubile’s study, we paid much attention to the process of sesquiterpenoid and triterpenoid biosynthesis (KO:zma00909). Expression levels of GRMZM2G091456 and GRMZM2G105787 genes for squalene epoxidases were greatly increased in maize kernels at 10 dpi (late) as well as Tps6 gene at 1 dpi (early), indicating that macrocarpene and triterpenoid may accumulate higher levels in maize seeds after *F. verticillioides* infection. In our dataset, large numbers of tropine and hydrogen cyanide synthesis-related genes were upregulated after *F. verticillioides* infection. A recent study showed that levels of cyanide are closely related with the capability of disease resistance in *Arabidopsis* (Rajniak et al., 2015). These results provide us a comprehensive insight into the resistance mechanisms of maize to *F. verticillioides* meanwhile give rise to a warning that endogenous maize antimicrobial substances may be another security risk besides mycotoxin when *F. verticillioides* infection occurs in corn.

### Induced Systemic Acquired Resistance to *F. verticillioides* by Both PTI and Hormone Signaling Pathways

Plants are continuously exposed to diverse phytopathogenic microorganisms and have elaborated a variety of defense mechanisms to successfully avoid infection by limiting pathogen invasion and multiplication (Glazebrook, 2005; Panstruga et al., 2009). Pathogens induce pathogen-associated molecular pattern (PAMP)-triggered immunity (PTI) and effector-triggered immunity (ETI) in plants. PTI is generally triggered by pathogen signals whereas ETI by resistance proteins that can recognize specific pathogen effectors. As the front line of plant defense against pathogenic microbes, PTI plays a critical role in resistance to environmental microbes (Chisholm et al., 2006). Correspondingly, a large number of genes involved in PTI signaling pathway were dramatically induced in BT-1 maize after *F. verticillioides* inoculation but not ETI genes, suggesting that PTI plays primary role in the resistance of BT-1 maize to *F. verticillioides*. This conclusion is further supported by the evidence of GO term functional analysis, in which DEGss between *F. verticillioides*-treated samples and pretreated or water-treated samples are significantly enriched in terms of secondary cell wall generation and hypersensitive reactions. Both two processes are typical responses in plant resistance induced by PTI pathway.

Hormone signaling plays crucial roles responding to biotic and abiotic stress in plants. The essential roles of SA and ET/JA-mediated signaling pathways in resistance to pathogens are well described (Robert-Seilaniantz et al., 2011). SA signaling positively regulates plant defense against biotrophic pathogens whereas ET/JA pathways are commonly required for resistance to necrotrophic pathogens and to herbivorous pests (Glazebrook, 2005; Bari and Jones, 2009). *F. verticillioides* is a facultative parasite pathogen, and there is no evidence that which hormone signaling pathways are particularly responded to this kind of fungal. Lanubile et al. (2014) reported that genes involving in ET/JA signaling pathways were significantly induced in the resistant material of CO441 compared with the susceptible material of CO354 after *F. verticillioides* inoculation. However, the genes involving in SA signaling pathway remained unchanged. Based on our data presented here, many genes associated with JA or SA signaling pathway were found to be increased in *F. verticillioides*-treated sample compared with pretreatment or water-treatment sample. This variation is likely the reasons of the differences in material, pathogenic races, and pathogen inoculation ways. Additionally, the plant hormone of ABA, originally described for their function in response to abiotic stresses, has recently emerged as crucial players in plant–pathogen interactions (Ton et al., 2009; Mang et al., 2012). After *F. verticillioides* inoculation, transcript levels of genes associated with ABA signaling pathway were dramatically induced in BT-1 maize, and the similar express trend was also observed in the susceptible maize of N6. Collectively, these results demonstrate that the resistant characteristic of BT-1 maize to *F. verticillioides* is achieved somehow through PTI-induced acquired systemic immunity as well as representative hormone signaling pathways.

### The Resistance to *F. verticillioides* May Be the Consequence of Minor Genes in BT-1 Maize

Extensive studies of ear rot resistance in maize have identified many related QTLs as well as highly resistant materials (Pérez-Brito et al., 2001; Robertson-Hoyt et al., 2006; Zhang et al., 2006; Ding et al., 2008; Xiang et al., 2010; Li et al., 2011; Chen et al., 2012). To data, however, any of genes or QTLs related to ear rot resistance has not been cloned or verified in maize, respectively. Thus we propose that corn resistance to ear rot by *F. verticillioides* is not controlled by major genes but a set of minor genes. This speculation is supported at least four lines of evidence. First, our analyses show that the high resistance of BT-1 maize to *F. verticillioides* is through PTI-induced systemic acquired immunity response, in which multiple genes are involved. Second, BT-1 also displayed various resistant performances of grain rot, stalk rot, seedling blight, and rust disease besides ear rot resistance (Chen et al., 2002; Liu et al., 2005). Third, multiple hormone signaling pathways comprising large number of *F. verticillioides* induced genes, such as ABA, SA, and JA, were involved in the resistance of BT-1 maize. Furthermore, DEGss responsive to *F. verticillioides* inoculation either at 1 or 10 dpi can be mapped to known QTLs related to ear rot resistance. Most of these detected QTLs contain a cluster of genes. This may partially explain the most common disability during positional cloning of QTLs for ear rot resistance, such as frequent changes of located regions in near isogenic lines (NIL) and degeneration of QTL effects along with NIL genetic background recovering.

It is worth noting the variations in mapping to known QTLs with DEGss between 1 and 10 dpi samples, which is considered the reason of differences in the way that QTLs function. Interestingly, this discrepancy consistent with the expression patterns of several genes in the representative pathways. For instance, numbers of genes responsive to *F. verticillioides* inoculation at both early and late stages are preferentially enriched the 177,381,672–193,733,670bp region of chromosome 3. Twelve gene models of *GRMZM2G007286. GRMZM2G349651*,*GRMZM2G003385, GRMZM2G563130, GRMZM2G589052, GRMZM2G511083, GRMZM2G305783, GRMZM2G506022, GRMZM2G092474, GRMZM2G310368, GRMZM2G355326*, and *GRMZM2G515027* are annotated in this region. Of these annotated genes, 4 and 6 genes were specifically regulated in response to *F. verticillioides* infection at 1 and 10 dpi, respectively. The other two genes of *GRMZM2G007286* and *GRMZM2G511083* were responsive to *F. verticillioides* infection at both 1dpi and 10dpi in our dataset (**Supplementary Figure S9**). Therefore, it is speculated that this QTL functions through the whole process of invasion and expansion of *F. verticillioides* in maize. A similar case is the 11,675,739–15,281,332 bp region of chromosome 3, which includes a QTL potential for early resistant response (**Supplementary Figure S9A**). In addition, another interesting region is located between 150,719,642 and 155,178,165 bp on the chromosome 6, which comprises *GRMZM2G047677* gene associated with ABA signaling pathway and one possible QTL for late resistant response at 10 dpi (**Figure 6**; **Supplementary Figure S9B**). Of cause, this does not really provide convincing final results because that some QTL regions are usually large to very large due to missing recombination in the centromeric regions and uncertainties in QTL mapping. The classification results of these QTLs are detailed in the **Supplementary Table S3**.

Collectively, our results suggest that the analysis of cooperative effect of genes would provide more knowledge on understanding the molecular mechanisms for ear rot disease resistance in corn. An exciting possibility that needs to be further explored is that sHSP proteins as well as representative secondary metabolites and signal transductions in current work may directly regulate the resistance of maize to *F. verticillioides*.

## Author Contributions

Project design: YW, ZZ, ZX, HZ, and JW. Data cultivation and collection: YW, ZZ, and YW. Data analysis: YW, ZZ, JG, and HZ. qPCR verification: JG. Writing: YW, ZZ, HZ, and JW.

## Conflict of Interest Statement

The authors declare that the research was conducted in the absence of any commercial or financial relationships that could be construed as a potential conflict of interest.

## Footnotes

1 http://www.ebioservice.com

2 ftp://ftp.ensemblgenomes.org/pub/plants/release-25/fasta/zea_mays

## Supplementary Material

**FIGURE S1 | The parallel test with microarray hybridizations.** **(A)** The sample before treatment. **(B)** The sample 1 day post inoculation by *F. verticillioides*. **(C)** The sample 10 days post inoculation by *F. verticillioides*. FPKM, the FPKM of genes by RNA-seq. NOR, the normalized expression of single-probe genes by microarray hybridizations.

**FIGURE S2 | The specifically regulated genes in the tropine biosynthesis pathway.**

**FIGURE S3 | The specifically regulated genes in the hydrogen cyanide biosynthesis.**

**FIGURE S4 | The specifically regulated genes in the phenylalanine metabolism pathway.**

**FIGURE S5 | The specifically regulated genes in the sesquiterpenoid and triterpenoid biosynthesis processes.**

**FIGURE S6 | The specifically regulated genes in the protein processing in the endoplasmic reticulum pathway.**

**FIGURE S7 | The specifically regulated genes in the plant hormone signal transduction.**

**FIGURE S8 | The specifically regulated genes in the plant-pathogen interaction process.**

**FIGURE S9 | Distribution of DEGs located in the metaQTL for *Fusarium verticillioide* ear rot.** **(A)** DEGs at 1 dpi were showed on the metaQTL map. **(B)** DEGs at 10 dpi were showed on the metaQTL map.

**TABLE S1 | Expression levels and annotation results of specifically expressed genes after *F. verticillioides* inoculation.**

**TABLE S2 | Expression levels and annotation results of specifically regulated genes response to *F. verticillioides* inoculation at 1pdi and 10pdi.**

**TABLE S3 | The classification of metaQTLs for maize ear rot.**
