# Supplementary figures and images for: The Mechanisms of Maize Resistance to Fusarium verticillioides by Comprehensive Analysis of RNA-seq Data
Source: Front Plant Sci. 2016 Nov 4;7:1654. doi: 10.3389/fpls.2016.01654 (PMC5096342; doi:10.3389/fpls.2016.01654)

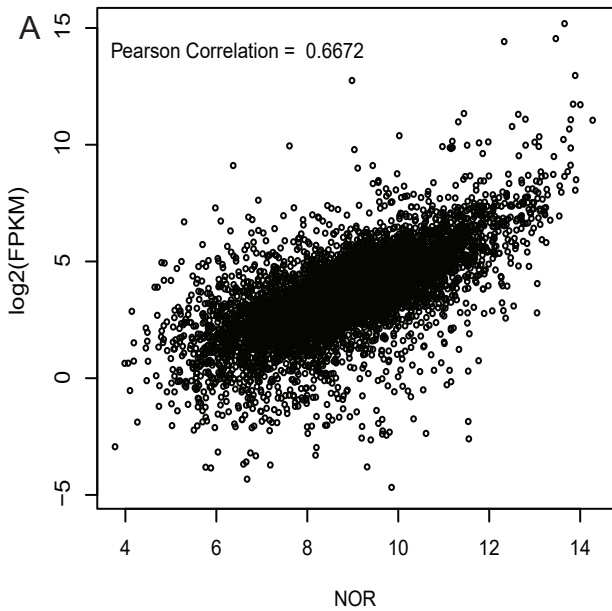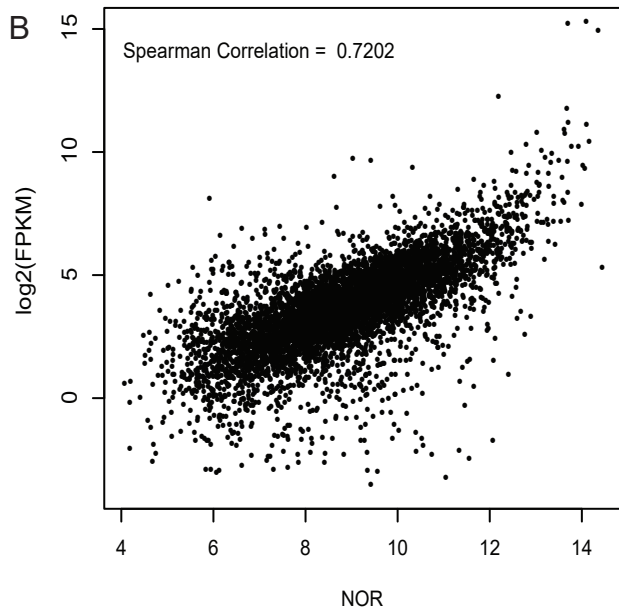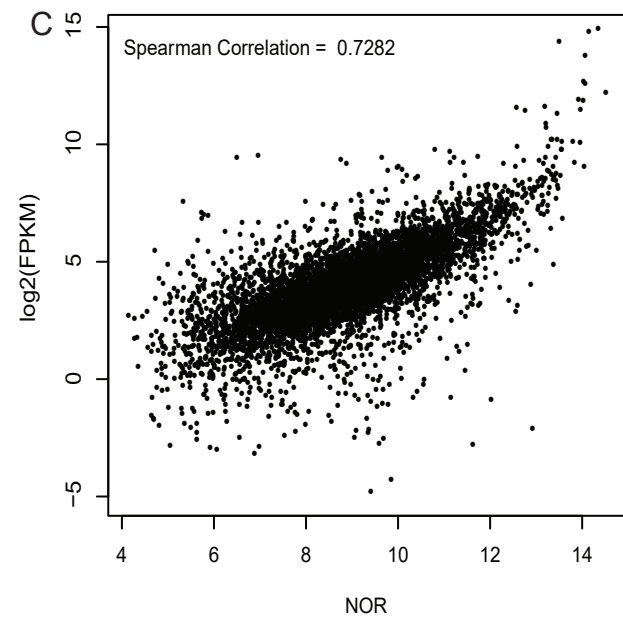

Supplement: Supplementary file 1 [file Image_1.PDF]

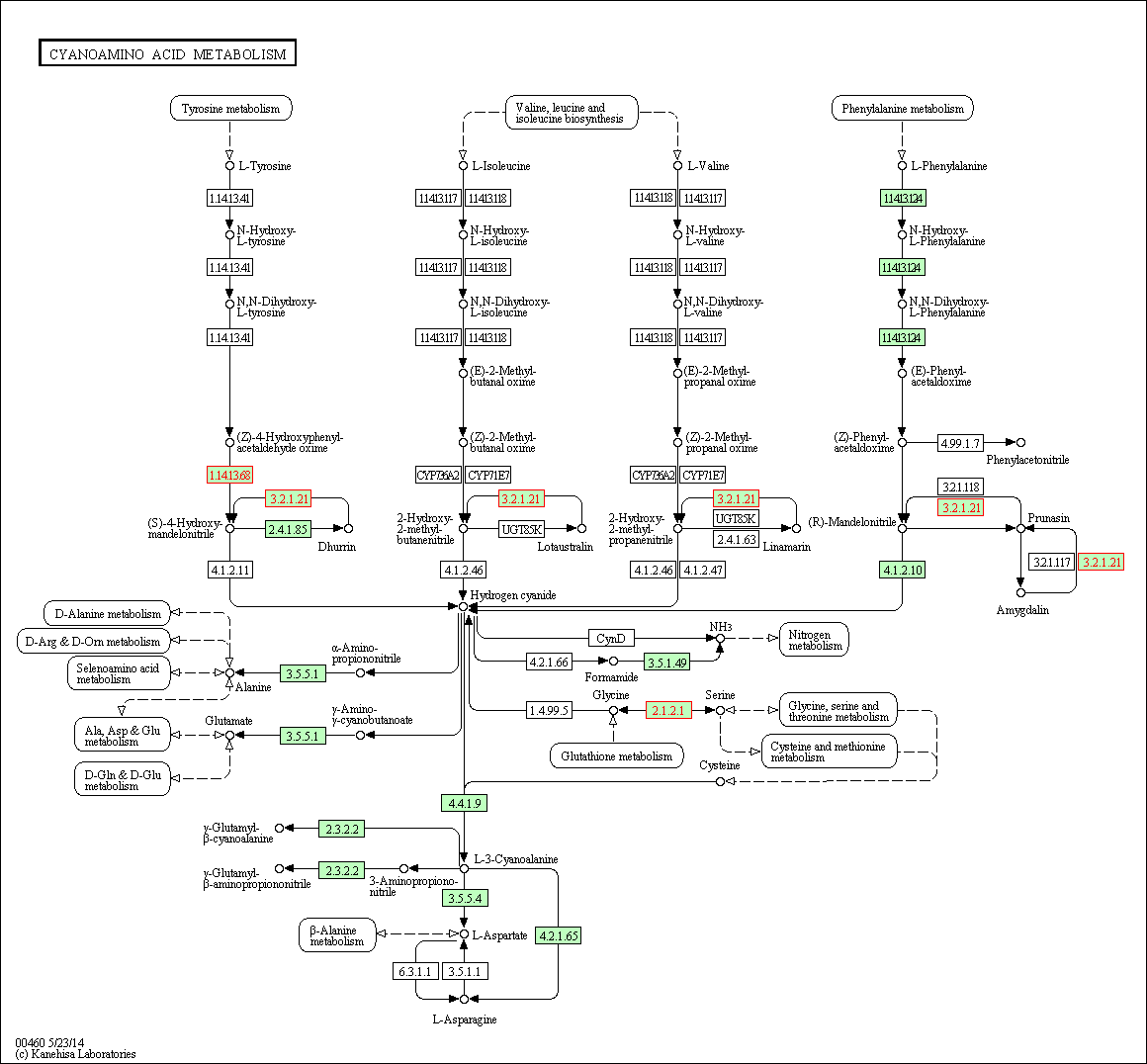

Supplement: Supplementary file 2 [file Image_2.PNG]

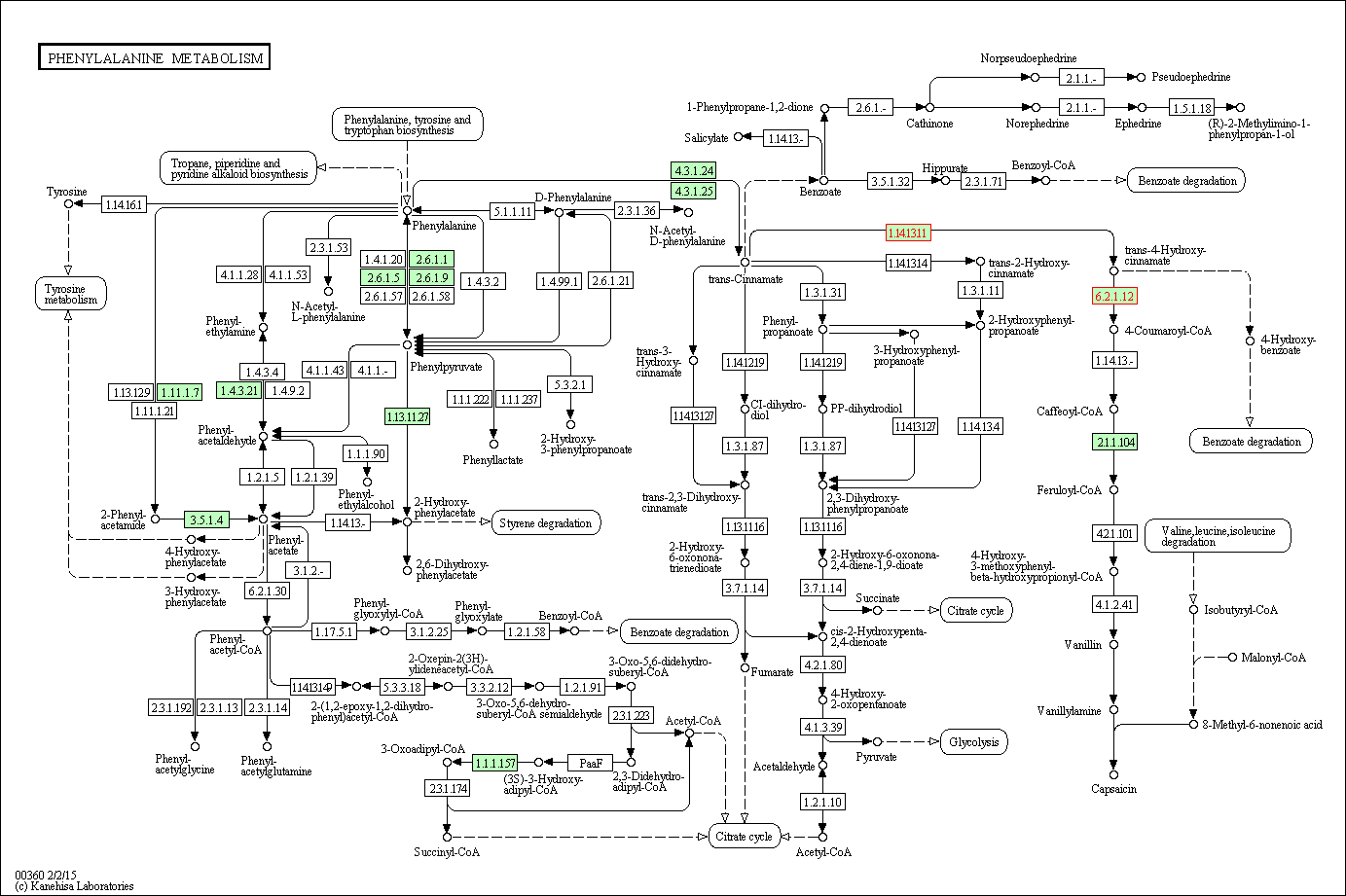

Supplement: Supplementary file 4 [file Image_4.PNG]

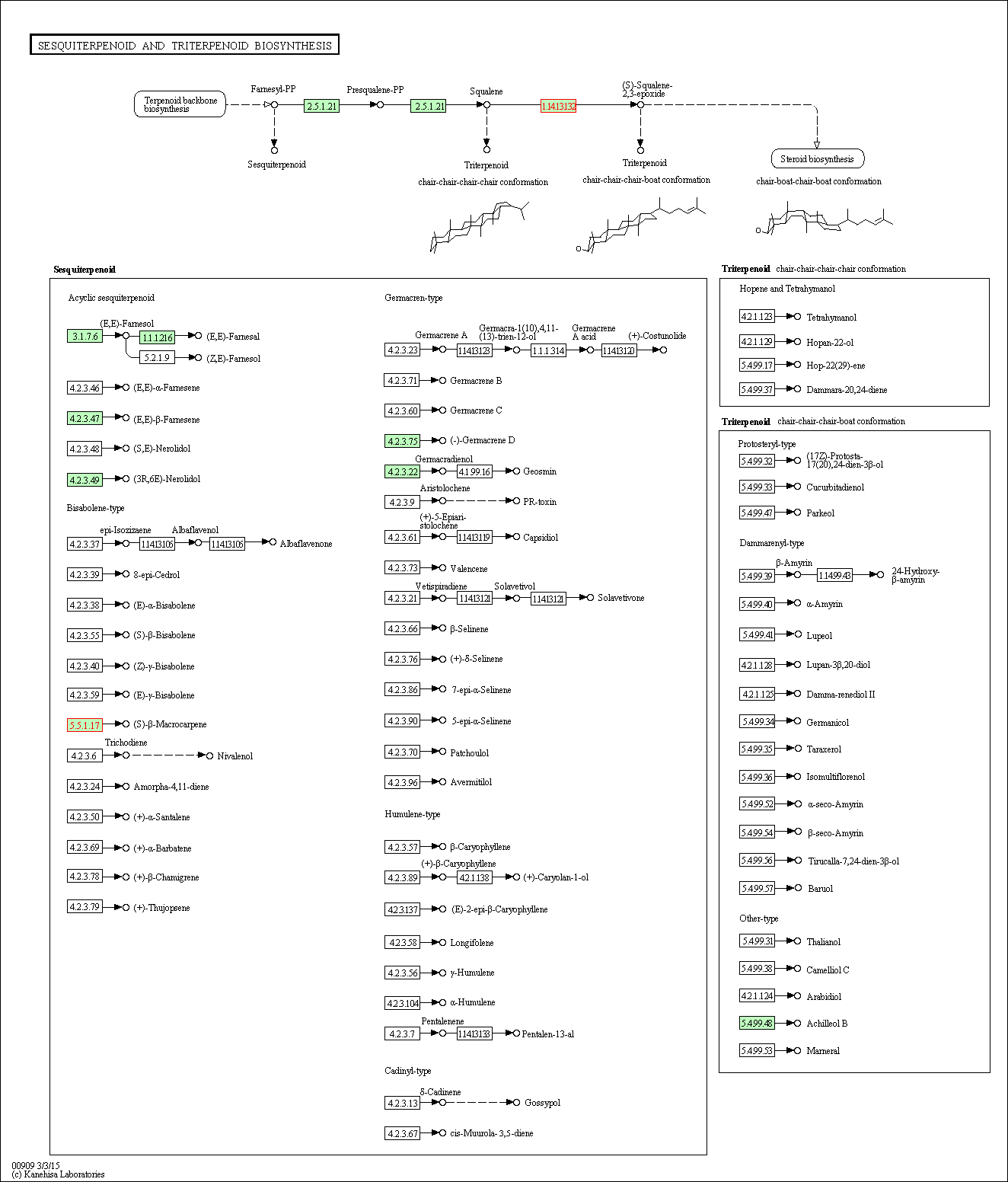

Supplement: Supplementary file 5 [file Image_5.PNG]

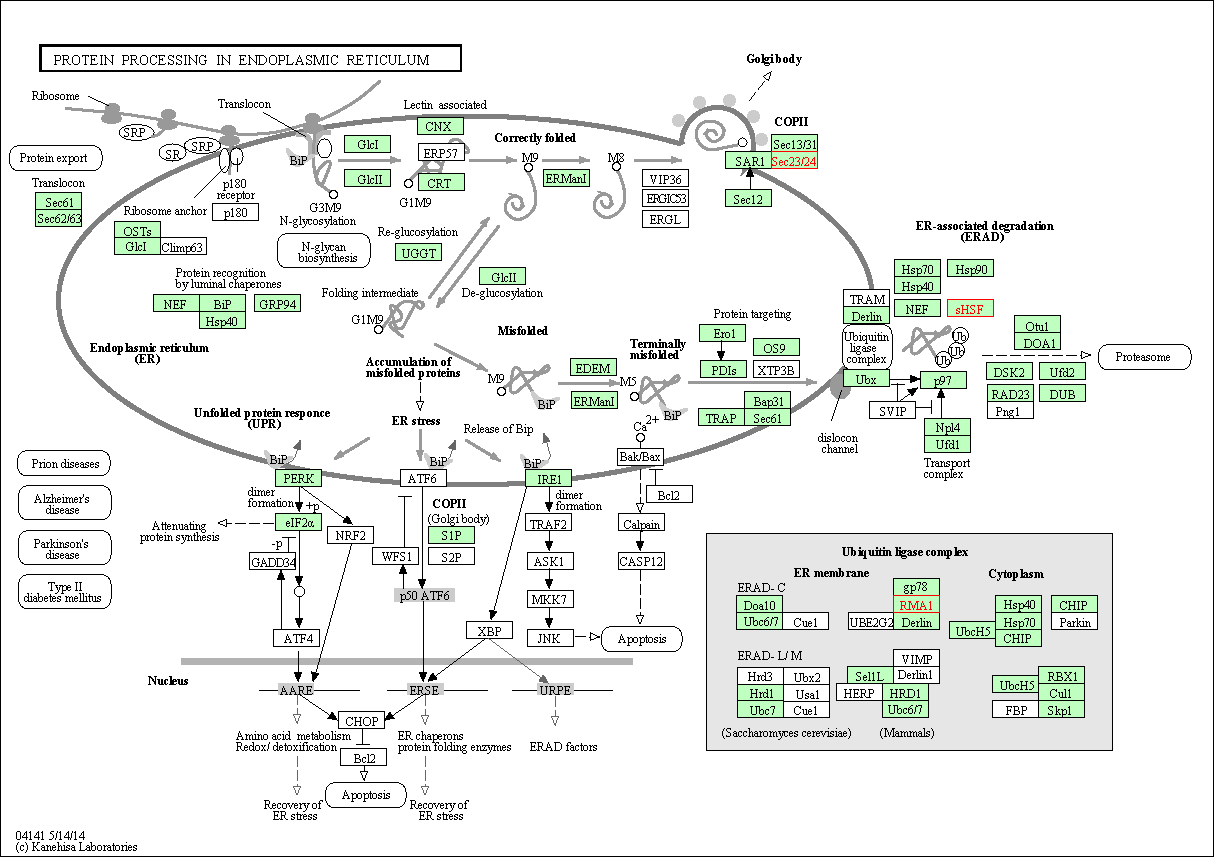

Supplement: Supplementary file 6 [file Image_6.PNG]

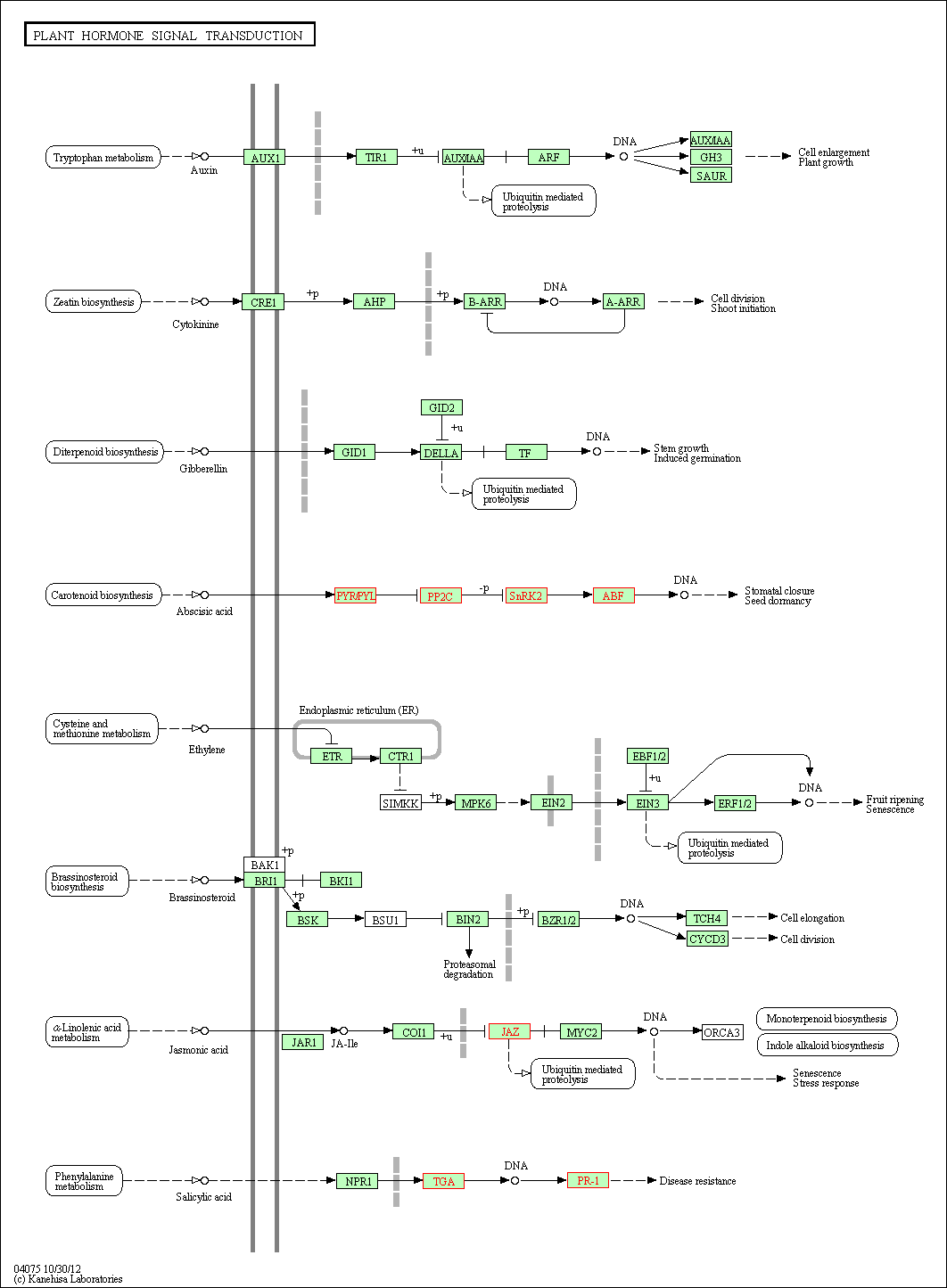

Supplement: Supplementary file 7 [file Image_7.PNG]

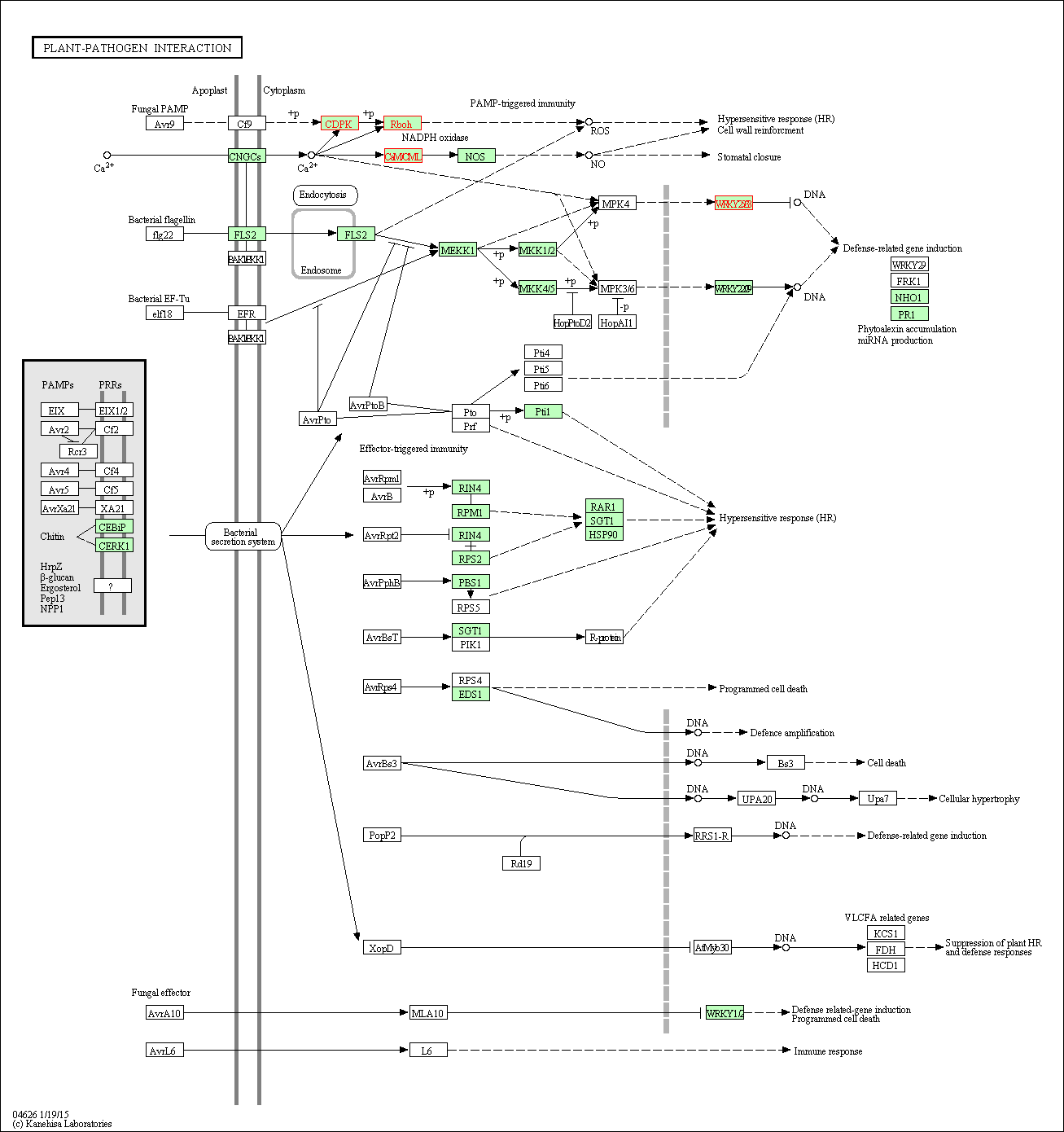

Supplement: Supplementary file 8 [file Image_8.PNG]

A

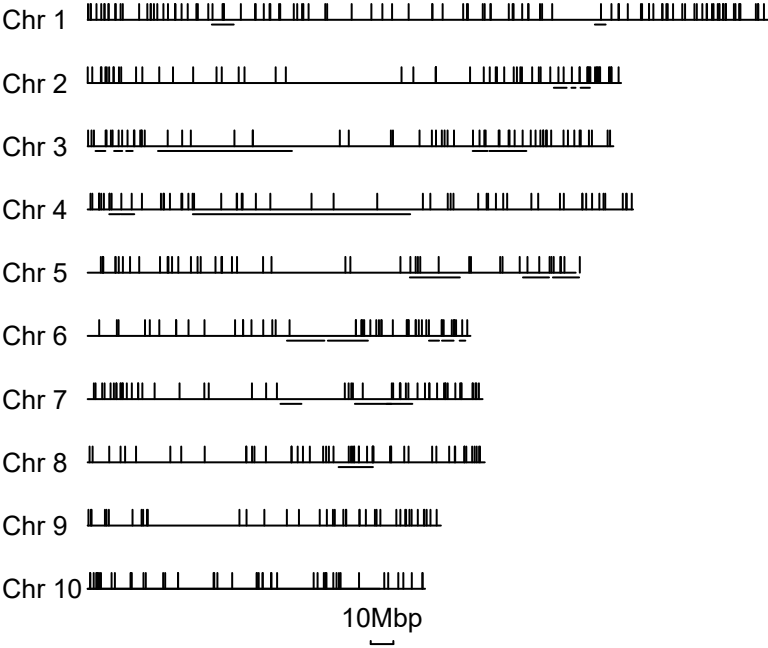

B

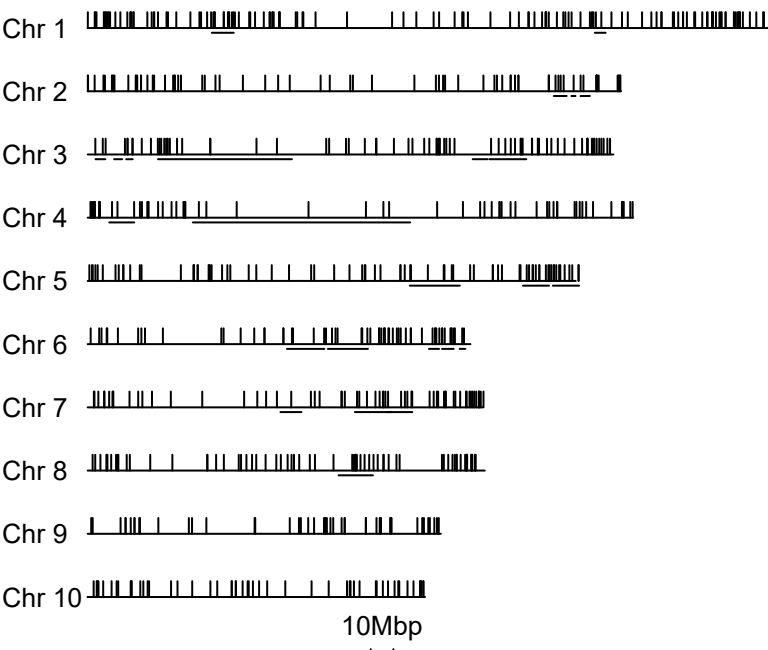

Supplement: Supplementary file 9 [file Image_9.PDF]
